# Can environment have an impact on health and wellbeing outcomes? Preliminary research on two Italian care homes for people with dementia

**DOI:** 10.1002/alz70858_106429

**Published:** 2025-12-26

**Authors:** Silvia Mangili

**Affiliations:** ^1^ Politecnico di Milano, Milano, Italy

## Abstract

**Background:**

The global prevalence of dementia is continually rising and is expected to double in the coming years. This demographic shift underscores the need for care facilities that prioritize the health and quality of life (QOL) of people with dementia (PWD). While the built environment (BE) significantly impacts user well‐being, holistic research in this area remains scarce.

**Method:**

This study explores the correlations between BE quality and PWD health outcomes. A validated evaluation tool was used to assess two facilities, followed by an analysis of clinical data from 20 PWD.

**Result:**

Results revealed that residents in the higher‐rated facility (S2) experienced improvements in QOL and depression compared to those in the lower‐rated facility (S1). Cognitive decline, daily living activities, and fall rates decreased similarly across both facilities.

**Conclusion:**

These findings highlight the importance and implication of BE in health and wellbeing of PWD, with future studies aiming for larger sample sizes for robust conclusions.

